# Atopic Dermatitis During Ustekinumab Therapy in a Patient With Ulcerative Colitis: A Case Report and Review of Targeted Therapies in Coexisting Diseases

**DOI:** 10.7759/cureus.108859

**Published:** 2026-05-14

**Authors:** Aya Maeyama, Takuya Inoue, Kazunari Sugita

**Affiliations:** 1 Internal Medicine, Faculty of Medicine, Saga University, Saga, JPN

**Keywords:** atopic dermatitis, biologics, jak inhibitors, ulcerative colitis, ustekinumab

## Abstract

Atopic dermatitis (AD) and ulcerative colitis (UC) are immune-mediated inflammatory diseases that may coexist, creating therapeutic challenges during biologic treatment. Here, we report a patient with long-standing AD and UC who was receiving ustekinumab therapy for UC and subsequently developed worsening eczema. In such cases, distinguishing an AD flare from a drug-induced eruption or secondary infection is a key clinical challenge because this distinction directly influences decisions regarding continuation or discontinuation of biologic therapy. Clinicopathologic evaluation supported the diagnosis of an AD flare rather than a drug-induced reaction. Temporary discontinuation followed by resumption of ustekinumab did not result in recurrence of cutaneous lesions, and the patient’s UC remained stable. In addition, we conducted a focused review of previously reported cases of patients with concurrent AD and UC treated with biologics or Janus kinase (JAK) inhibitors and summarized their clinical characteristics and outcomes. This report highlights clinical considerations when selecting biologic or targeted therapies in patients with coexisting AD and UC and underscores the importance of careful clinicopathologic assessment of cutaneous eruptions during biologic therapy.

## Introduction

Biologic therapies are increasingly used in the management of immune-mediated inflammatory diseases, including atopic dermatitis (AD). AD is primarily driven by Th2-dominant inflammation, whereas ulcerative colitis (UC) is more closely associated with Th17/IL-23-mediated immune responses, although Th2-related cytokines may also contribute in certain contexts [1]. These partially distinct yet overlapping immune pathways may influence therapeutic responses when these conditions coexist.

In this setting, the development or exacerbation of cutaneous eruptions during biologic therapy presents a diagnostic and therapeutic challenge. In particular, distinguishing an AD flare from a drug-induced eruption or secondary infection can be difficult, yet this distinction is critical for appropriate clinical decision-making. Patients with both AD and UC represent a particularly challenging group because biologic therapies used for one condition may influence the activity of the other. Several reports have described the use of biologics or Janus kinase (JAK) inhibitors in patients with concurrent AD and UC, but clinical experience remains limited [2-6].

To our knowledge, reports describing patients with concurrent AD and UC receiving ustekinumab are extremely limited, and the clinical course of AD during ustekinumab therapy in this comorbid setting has not been well characterized. Here, we present a case of AD in a patient with UC receiving ustekinumab therapy and review previously reported cases of AD and UC treated with biologics or JAK inhibitors to better understand therapeutic considerations in this comorbid population.

## Case presentation

A 26-year-old male with a history of AD since childhood and UC diagnosed at 18 years of age was referred to our department for worsening eczema. He had been receiving ustekinumab (initial 260 mg intravenous induction dose followed by 90mg subcutaneous injections every eight weeks) for UC for two years and was also treated with oral olopatadine hydrochloride. Despite treatment with topical corticosteroids and antihistamines, his AD remained poorly controlled.

**Figure 1:**
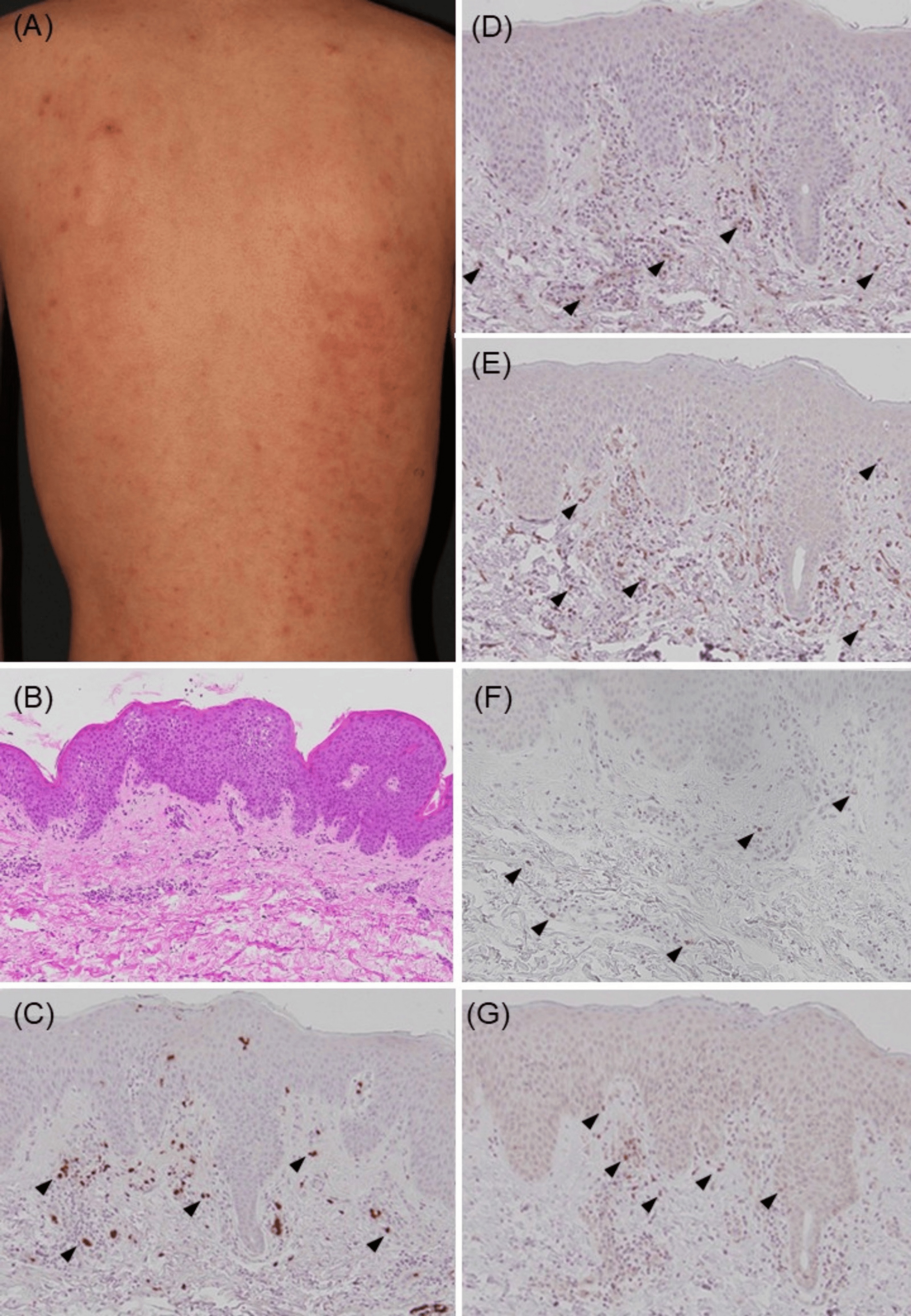
Clinical and histopathologic findings in AD and UC (A) Clinical findings showing erythema, papules, and excoriations on the back. (B) Skin biopsy of the back (H&E staining) showing mild epidermal thickening, elongated rete ridges, spongiosis, and infiltration by neutrophils, histiocytes, and lymphocytes. (C-G) Immunohistochemical staining of the same biopsy specimen: neutrophil elastase (C), CD68 (D), CD163 (E), MMP-12 (F), and IL-1β (G). Arrowheads indicate representative areas of staining. (C) Neutrophil elastase highlights neutrophils in both the epidermis and dermis, indicating innate immune activation. (D, E) CD68 and CD163 mark dermal macrophages; CD68 is a pan-macrophage marker, whereas CD163 is associated with alternatively activated or regulatory macrophages, which are often observed in chronic inflammation and tissue remodeling in AD. (F) MMP-12, a matrix metalloproteinase produced mainly by macrophages, is involved in extracellular matrix degradation and immune cell trafficking; it is upregulated in AD and is considered a marker of macrophage-driven inflammation. (G) IL-1β is a proinflammatory cytokine implicated in epidermal barrier dysfunction and keratinocyte activation in AD; its expression in both epidermal and dermal compartments suggests active innate immune signaling. H&E, hematoxylin and eosin; MMP12, matrix metalloproteinase-12; IL-1β, interleukin-1β; AD, atopic dermatitis; UC, ulcerative colitis

Physical examination revealed erythematous papules, excoriations, and marked xerosis involving the face, trunk, and lower extremities (Figure 1A). A skin biopsy obtained from the back demonstrated mild epidermal hyperplasia, elongation of the rete ridges, spongiosis, and perivascular lymphocytic infiltration in the upper dermis, findings consistent with AD (Figure 1B). In addition, scattered neutrophils and histiocytes were observed within the dermal infiltrate.

Immunohistochemical staining was positive for neutrophil elastase, CD68, and CD163, confirming the presence of neutrophils and macrophages (Figure 1C, 1D, 1E). Additional staining for matrix metalloproteinase-12 (MMP-12) and interleukin (IL)-1β showed positivity in the dermis (Figure 1F, 1G). These findings indicated active cutaneous inflammation with involvement of innate immune cells, a pattern that has been increasingly recognized during inflammatory flares of AD, although it is not specific to a single inflammatory dermatosis [7,8]. This pattern supports inflammatory changes consistent with an AD flare rather than a drug-induced eruption.

Laboratory evaluation revealed elevated serum IgE levels (1,840 IU/mL; normal: <170 IU/mL) and thymus and activation-regulated chemokine (TARC) levels (5,400 pg/mL; normal: <450 pg/mL). Complete blood counts, liver and renal function tests, and C-reactive protein (CRP) levels were within normal limits. Because a drug-induced eruption could not initially be excluded, ustekinumab was temporarily discontinued. Empirical antibiotic therapy was initiated but failed to produce clinical improvement.

In this case, potential exacerbating factors included psychological stress, sleep disturbance, and suboptimal adherence to topical therapy. A drug-induced eruption was considered in the differential diagnosis because the distribution and morphology of the skin lesions initially raised suspicion. However, the clinical course, treatment response, and lack of temporal association with drug administration made a drug-induced allergic reaction unlikely. Infectious causes were excluded based on normal laboratory findings, including white blood cell count and CRP levels. Although clinicopathological findings and the response to topical therapy suggested an AD flare, a drug-induced eruption could not be definitively excluded at that stage.

Ustekinumab was resumed three weeks after discontinuation, and no recurrence or exacerbation of skin lesions was observed, supporting the interpretation that a drug-induced eruption was unlikely. As his condition stabilized, topical difamilast monotherapy was initiated, while oral olopatadine hydrochloride was continued. Over the subsequent year, serum IgE and TARC levels decreased to 1,370 IU/mL and 665 pg/mL, respectively, and the patient remained relapse-free for 18 months. In our case, serum IgE and TARC levels decreased in parallel with clinical improvement; however, these markers should be interpreted with caution, as they are not disease-specific and may be influenced by multiple factors.

## Discussion

Autoimmune comorbidities such as UC are increasingly recognized in patients with AD. Epidemiologic studies have demonstrated that the risk of UC is higher in individuals with moderate-to-severe AD, suggesting shared immune dysregulation between these conditions [9]. Consequently, clinicians are increasingly faced with therapeutic decisions that must balance disease control and safety across multiple immune-mediated disorders.

To better understand treatment strategies in this setting, we reviewed previously reported cases of patients with concurrent AD and UC treated with biologics or JAK inhibitors (Table 1). Six reported cases, including the present case, have described the use of biologics or JAK inhibitors in patients with concurrent AD and UC. Across these cases, clinical outcomes varied according to the therapeutic target. Dupilumab, an inhibitor of the IL-4/IL-13 pathway, was associated with improvement or stability of AD in several patients [2]. However, exacerbation of UC during dupilumab therapy was observed in at least one reported case, necessitating treatment discontinuation and a switch in therapy [3]. These observations are consistent with emerging reports suggesting that blockade of type 2 cytokines may, in some patients, unmask or exacerbate underlying intestinal inflammation [10].

**Table 1:** Clinical characteristics of patients with concurrent AD and UC treated with biologics or JAK inhibitors AD, atopic dermatitis; UC, ulcerative colitis; JAK, Janus kinase

Case (author, year)	Age/sex	Duration of AD	Duration of UC	Biologic/JAK inhibitor	Clinical course after biologic	Action taken	Outcome	Notes
Gracia-Darder et al. [2], 2022	28/F	27 years	>4 years	Dupilumab	AD: Improved; UC: Remained stable	Continued	Both AD and UC remained stable	Continued during pregnancy, with stable skin symptoms
Seishima et al. [3], 2023	20/F	Since early childhood	UC onset 4 months after initiation of dupilumab	Dupilumab; upadacitinib	AD: Improved; UC: Exacerbated by dupilumab; improved with upadacitinib	Dupilumab discontinued; upadacitinib initiated	Both AD and UC remained stable	
Grieco et al. [4], 2023	36/M	Not available	1 year	Upadacitinib	AD: Improved; UC: Improved	Continued	Both AD and UC remained stable	
Giovanni Lasagni et al. [5], 2024	31/F	Since infancy	10 years	Dupilumab; vedolizumab; upadacitinib	AD: Temporarily improved, then worsened with dupilumab. Remission maintained with upadacitinib. UC: In remission with mesalazine and vedolizumab. Remission maintained with upadacitinib.	Following discontinuation of dupilumab and vedolizumab, upadacitinib was administered	AD achieved near-complete remission, except for facial erythema. UC remained stable.	
Sailish Honap et al. [6], 2021	46/M	46 years	20 years	Tofacitinib	AD: Improved; UC: Improved	Continued	Both AD and UC remained stable	Also effective for alopecia universalis
Our case	26/M	Since childhood	8 years	Ustekinumab	AD flare after 2 years of UC therapy	Temporarily discontinued; resumed after 3 weeks.	UC remained stable, AD was controlled with topical therapy	AD flare was likely due to poor adherence and stress; no recurrence after resumption

In contrast, JAK inhibitors such as upadacitinib and tofacitinib were associated with improvement or stable disease in both AD and UC across multiple cases [3-6]. Given their broader suppression of downstream cytokine signaling, JAK inhibitors may offer therapeutic advantages in patients with overlapping inflammatory pathways affecting both the skin and gastrointestinal tract. These findings underscore the importance of considering the dominant inflammatory drivers of each comorbid condition when selecting targeted therapy. In this context, upadacitinib was also considered a potential therapeutic option. However, in our case, because UC was well controlled with ustekinumab at the time of presentation, switching systemic therapy was not considered necessary.

Ustekinumab, a monoclonal antibody targeting the IL-12/23p40 subunit, is widely used for inflammatory bowel disease, including UC. However, its efficacy in AD has been inconsistent in previous reports. Current evidence regarding the efficacy of ustekinumab in AD remains limited and heterogeneous; although some reports suggest clinical improvement, overall outcomes have been inconsistent, and the available data are insufficient to establish its role in AD management [11,12]. Reports describing patients with concurrent AD and UC receiving ustekinumab are limited, and the clinical course of AD in this setting has not been well characterized. In the present case, ustekinumab had been administered for UC for two years before the patient developed worsening eczema. Importantly, the flare responded promptly to topical therapy, and resumption of ustekinumab did not result in recurrence of skin lesions while UC remained stable. This observation supported the diagnosis of an AD flare, as the absence of recurrence after the reintroduction of ustekinumab made a drug-induced eruption unlikely.

Histopathologic and immunohistochemical findings provided supportive information for clinicopathologic correlation. The identification of neutrophils and macrophages within the dermal infiltrate was interpreted as reflecting active inflammatory changes compatible with an AD flare rather than an alternative dermatosis or drug-induced eruption [13]. These findings were considered supportive of clinicopathologic correlation but did not independently guide therapeutic decision-making.

Taken together with the reviewed cases, our findings highlight the complexity of selecting targeted therapies in patients with comorbid AD and UC. Careful assessment of disease activity in both organs and individualized therapeutic decisions are essential when managing such patients. Importantly, this case and the reviewed literature emphasize that cutaneous exacerbations occurring during biologic therapy in patients with concurrent AD and UC should not automatically be attributed to drug-related adverse events, and careful clinicopathologic evaluation is essential before discontinuing effective systemic therapy.

## Conclusions

This report describes a patient with concurrent AD and UC who experienced an AD flare during ustekinumab therapy for UC. Together with our review of previously reported cases treated with biologics or JAK inhibitors, this case highlights the therapeutic challenges involved in managing patients with comorbid AD and UC. The findings underscore the importance of carefully evaluating cutaneous eruptions that occur during biologic therapy in order to distinguish disease flares from drug-induced reactions. Such assessment may help guide individualized treatment decisions when managing patients with overlapping immune-mediated diseases. This observation supported the diagnosis of an AD flare, as the absence of recurrence after the reintroduction of ustekinumab made a drug-induced eruption unlikely.
